# Deep reinforcement learning stock market trading, utilizing a CNN with candlestick images

**DOI:** 10.1371/journal.pone.0263181

**Published:** 2022-02-18

**Authors:** Andrew Brim, Nicholas S. Flann

**Affiliations:** Department of Computer Science, Utah State University, Logan, Utah, United States of America; Universidad Veracruzana, MEXICO

## Abstract

Billions of dollars are traded automatically in the stock market every day, including algorithms that use neural networks, but there are still questions regarding how neural networks trade. The black box nature of a neural network gives pause to entrusting it with valuable trading funds. A more recent technique for the study of neural networks, feature map visualizations, yields insight into how a neural network generates an output. Utilizing a Convolutional Neural Network (CNN) with candlestick images as input and feature map visualizations gives a unique opportunity to determine what in the input images is causing the neural network to output a certain action. In this study, a CNN is utilized within a Double Deep Q-Network (DDQN) to outperform the S&P 500 Index returns, and also analyze how the system trades. The DDQN is trained and tested on the 30 largest stocks in the S&P 500. Following training the CNN is used to generate feature map visualizations to determine where the neural network is placing its attention on the candlestick images. Results show that the DDQN is able to yield higher returns than the S&P 500 Index between January 2, 2020 and June 30, 2020. Results also show that the CNN is able to switch its attention from all the candles in a candlestick image to the more recent candles in the image, based on an event such as the coronavirus stock market crash of 2020.

## Introduction

Neural networks have proven successful in predicting financial markets. This includes the use of CNNs [1–4]. However the black box nature of neural networks creates strong demand for insight into how neural networks accomplish this. This work utilizes feature map visualization to generate a visual representation from the CNN weights to analyze how the neural network is able to do this [5–8]. The Google Brain Team DeepDream project has made recent advancements with feature map visualizations to understand neural networks. They claim these tools are one of the fundamental building blocks that will empower humans to understand neural networks [9]. In this work feature map visualizations are used to discover that a CNN can switch its attention from a wide focus of all the regions in an input image to a narrower focus, based on an event.

A specific type of reinforcement learning (RL) system, Double Deep Q-Network (DDQN), is used in this work since it has been shown to yield more accurate values and give better overall performance than other neural network systems [10]. Many recent applications have employed a DDQN as a result, including autonomous vehicles [11, 12], energy reduction and battery optimization [13–15], and robotics and UAV controls [16, 17].

Here a DDQN utilizing a CNN with only candlestick images as input can outperform the S&P 500 Index during one of the most unprecedented stock market crashes in financial history, the coronavirus stock market crash of 2020.

The motivation for this work is to bridge the gap in computer science research, and financial research, to test whether a RL system utilizing a CNN can outperform the S&P 500 Index. An additional motivation is to determine how the CNN predicts the stock market. There is existing research which trains a CNN to trade financial markets via images, including candlestick images, such as Tsai, Chen et. al 2019 [2] and Selvin, Menon et al 2017 [1]. There is also existing research which utilizes deep neural networks with reinforcement learning techniques for stock market predictions [18–20]. However no research has yet used feature map visualizations to reconstruct how the CNN predicts the stock market. Additionally there is no existing research which makes specific comparisons to the performance of financial markets, and comparisons to methodology of existing human based trading strategies.

Reinforcement learning is a artificial intelligence technique, where an agent interacts with an environment through actions. A state is provided by an environment, and the agent selects an action based on that state to maximize a reward. The agent learns through states and actions to maximize its reward [21]. In this work the agent is a DDQN, the state it receives is a candlestick image representing the previous 28 days of stock prices. The DDQN action is to take a long, short, or no position on the stock the next day.

**Fig 1:**
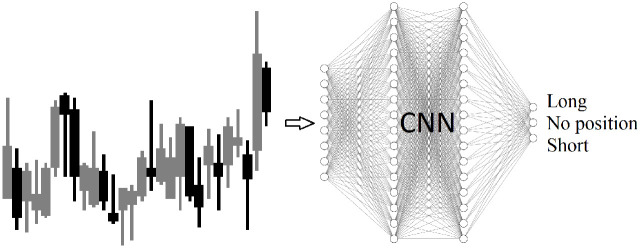
Candle stick images are generated based on the open, close, high, low prices for each day, for each stock. The CNN in the RL system receives candlestick images as input and outputs actions of long, short, or no position.

This work employs Q-learning, a type of temporal difference reinforcement learning, to approximate a policy function for each state in the space of trading parameters [22, 23]. Q-learning is combined with function approximation, utilizing a CNN to approximate a Q-function. An OpenAI Gym environment [24] is built to simulate the stock market and provide candlestick images to a CNN. The CNN outputs a long, short, or no position action, as shown in Fig 1. The action is sent to the environment. The environment returns the next state and a reward for the action taken.

A Deep Q network (DQN) is a multi-layered neural network that for a given input state, outputs a vector of action values [10]. This work employs a specific type of DQN, a Double Deep Q-Network, introduced by Google Deep Mind in 2016 [10] and utilized by the artificial intelligence AlphaGo which defeated the World Go champion Lee Sedol [25]. Van Hasselt, Guez, and Silver (2016), show that the idea behind the Double Q-learning algorithm (Van Hasselt, 2010), which was first proposed in a tabular setting, can be generalized to work with arbitrary function approximation, including deep neural networks. The Double DQN, not only yields more accurate value estimates, but leads to better overall performance of the deep neural network [10]. For this reason, an RL system utilizing a DDQN is chosen for this work. A CNN is used within the DDQN for function approximation, with candlestick images as input, and a trading position of long, short, or no position as output.

The DDQN is trained on candlestick images generated from stock market prices from 2013 through 2019. It is then tested on candlestick images generated from January 2, 2020 through June 30, 2020. The top 30 stocks in the S&P 500 Index are selected since the data is widely available, and this creates a large enough base of stocks to ensure robustness in results. The DDQN receives an image of 28 candlesticks for each day. This means for 30 stocks, there are a total of 52920 observations in the training data set, and 3780 observations in the testing data set. The training data set of seven years is selected to allow enough time for the DDQN to learn to trade each stock. This training data set also includes the China Tariffs Dispute stock market crash of 2018, which allows the DDQN to learn how to perform during a stock market crash. Six months is selected as the testing data set, allowing for enough time to verify the DDQN performance and also observe the behavior of the DDQN during the coronavirus stock market crash.

**Fig 2:**
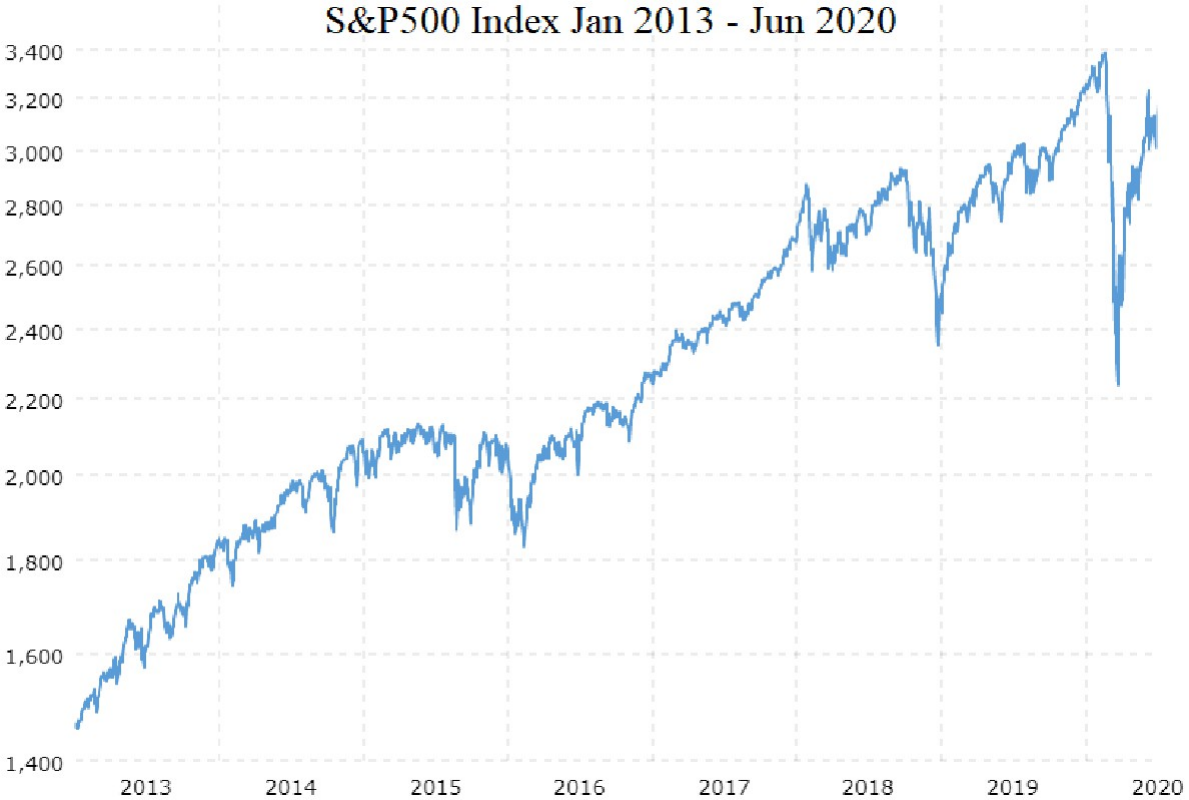
S&P 500 Index January 2013—June 2020. Training data consists of stock prices from January 2013 through December 2019. Testing data consists of stock prices from January 2020 through June 2020. The coronavirus stock market crash from Feb 20, 2020 to Mar 23, 2020 is a greater decline, 33%, than any event in the training data. The China tariffs dispute stock market crash in 2018 was closest, a 20.9% decline in 91 days.

The S&P 500 Index value dropped from $3372.23 to $2234.40 on Mar 23, 2020. A 33.7% loss in 31 days. The S&P 500 Index value began to quickly recover, increasing to $3232.39 on Jun 8, 2020. A 96% recovery in 45 days. The coronavirus stock market crash data is unlike any data in the training data set. The closest event in the training data set is the China tariff dispute stock market crash in 2018, where the S&P 500 declined 20.9% and subsequently recovered as shown in Fig 2.

Outperforming the S&P 500 Index, defined by this work, will be the DDQN yielding higher geometric returns than the S&P 500 Index for the testing data set. Tests are run with the largest 30 stocks of the S&P 500 including Apple Inc. (AAPL), Amazon.com (AMZN), Microsoft Corporation (MSFT), Google LLC (GOOGL), Facebook Inc. (FB), Adobe Inc. (ADBE), Berkshire Hathaway Inc. Class B (BRK.B), JPMorgan Chase Co. (JPM), Johnson Johnson (JNJ), Visa Inc. Class A (V), United Health Group Incorporated (UNH), Procter Gamble Company (PG), Walt Disney Company (DIS), Home Depot Inc. (HD), Mastercard Incorporated Class A (MA), Bank of America Corp (BAC), Exxon Mobil Corporation (XOM), Coca-Cola Company (KO), Intel Corporation (INTC), AT&T Inc. (T), Walmart Inc. (WMT), Boeing Airlines Co. (BA), Comcast Corporation (CMCSA), Cisco Systems Inc. (CSCO), Chevron Corporation (CVX), Merck and Company Inc. (MRK), PepsiCo Inc. (PEP), Pfizer Inc. (PFE), Verizon Communications Inc. (VZ), and Wells Fargo Co (WFC).

Human traders use candlestick images to make trading decisions based on calculable trends, but also experience. Candlestick images can allow the trader to see features that are not numeric. Feature map visualizations are used to interpret what in the input images is causing the DDQN to output a given action. The Google Brain Team research on feature visualization, specifically Olah, Mordvintsev, and Schubert (2017) [9] utilizes neural network weight reconstruction to generate images. They claim, in the quest to make neural networks interpretable, feature visualization stands out as one of the most promising and developed research directions.

**Fig 3:**
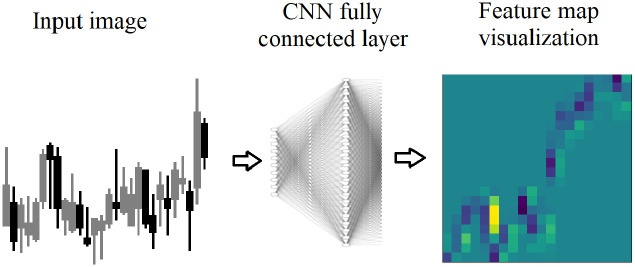
Feature map visualizations are generated from fully connected layers in the DDQN. A candlestick image is input, and each neuron in the fully connected layer receives the image. The neuron is excited on various parts of the image. The excited regions are stored as a 2D array, and shown here as a heatmap.

Feature map visualizations are generated from the input image and the regions of neuron excitement on the input image as show on the far right image in Fig 3. Neuron excitement is the activation output from the neurons, in a neural network. While there are multiple uses of feature map visualizations, this work will utilize the values of the feature maps output as a 2D array. A similar approach to Nguyen, Yosinski, Clune, et al. (2019) [8] is used in this work, because it provides the neuron excitement as 2D arrays that can be measured and analyzed.

The region in yellow indicates the highest neuron excitement, meaning the highest value output by the neuron. To generate a feature map visualization, after training, a new network is constructed with only two layers: an input layer for the input image, and a layer constructed from the weights of the fully connected layer. The input image is passed into the second layer and the neurons are excited according to their weights calculated during training, and the output is stored in a 2D array.

**Fig 4:**
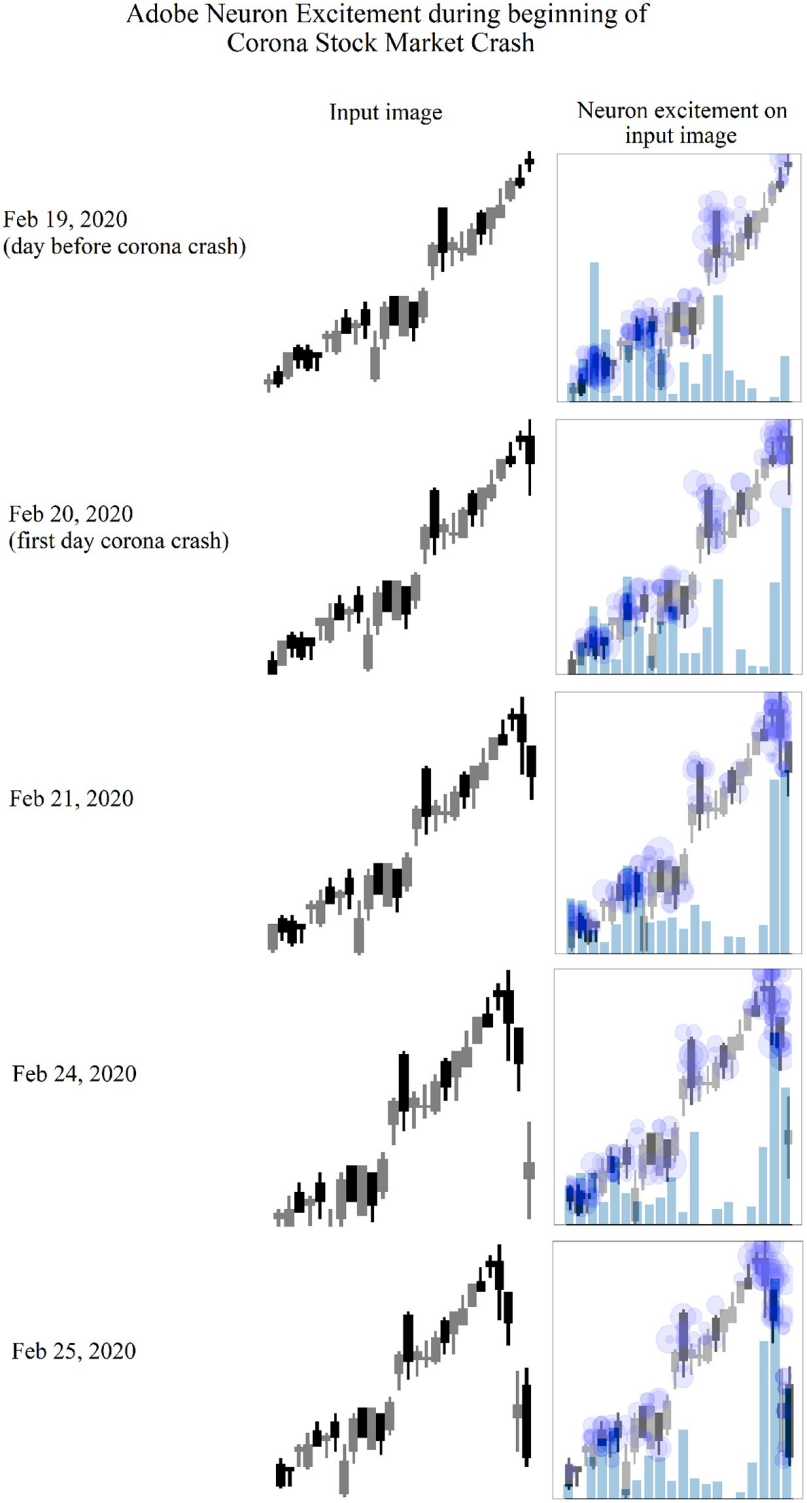
Adobe candlesticks images representing the day before, and the first four days of the coronavirus stock market crash. Each row represents the day the input image was supplied to the DDQN. The first column represents the input image. The second column displays the regions of the image excited by each neuron. The size of each blue dot represents the level of excitement. The blue is made darker by overlapping dots, indicating multiple neurons were excited by the same region of the image. The bars indicate the total excitement value.

Each candlestick image represents 28 days, while there are 20 regions generated in the feature map. This is due do the reshaping performed by the convolutional layer. Still, the 20 regions provide strong insight into which region provides the highest neuron excitement level, and which region of the candlestick image used by the DDQN to determine a trading signal. For example, ADBE candlestick images can be seen at the beginning of the corona stock market crash in Fig 4. As the stock market crash begins, the neuron excitement shifts from all 28 candlesticks to the most recent candlesticks. The day before the stock market crash, neuron excitement levels are highest on days 23 through 27, and day 9. However the next day, the first day of the crash, the neuron excitement level is highest on the most recent day of the candlestick image. The neuron excitement level is almost double the excitement level of any other day in the image. This excitement level continues on the most recent days as the coronavirus stock market crash continues.

While each of these feature map visualization tools are powerful in revealing how a CNN has learned, this work specifically needs to measure changes in neuron excitement. Therefore a similar approach to Nguyen, Yosinski, Clune, et al. (2019) is used in this work. To generate the output images shown in Fig 4, candlestick images are combined with the neuron activation output, or neuron excitement. The newly created image shows exactly which regions in the input image caused neuron excitement.

The contributions of this work is that it is the first to provide exploration of how a DDQN predicts the stock market, using feature map visualizations. This work also substantiates that artificial intelligent techniques, namely an RL system utilizing a DDQN, can be used to outperform the S&P 500 Index. It makes direct comparisons of the DDQN returns to financial market returns, where previous works have not.

## Background

In this section, existing literature is reviewed in two separate streams of research. First, the existing work in financial research on various trading strategies that are related to the tests proposed in this work. The associated profitability of these strategies documented in the literature is reviewed. Second, the prior work in the Computer Science literature that examines both deep learning and prediction in financial markets.

Previous work has shown that a CNN can be effective in stock market trading, but have not been applied with feature map visualization analysis, and have not compared their performance to the S&P 500 Index. In this work, feature map visualizations are used investigate the ability of a CNN to switch its attention from all days in a candlestick image, showing the full 28 day history, to the most recent days. This ability to switch from full history in a candlestick image, to the most recent days is different than existing technical trading indicators like those presented in Brock, Lakonishok and LeBaron (1992) and Skouras (2001). Marshall, Young and Rose (2006) show that candlestick patterns are not effective at predicting the stock market. However they use candlestick images between 1 and 5 days of price history, not 28 days like this RL system.

### Financial markets literature review: Price prediction and technical trading

In this section, the existing financial literature that examines technical trading strategies is reviewed. There is a long standing divide between financial fundamental analysis and technical trading analysis. Fama (1965), and also Fama (2021), argue that stock market prices are efficient [26, 27] and therefore technical trading indicators should not outperform the market. However, Brock, Lakonishok and LeBaron (BLL) (1992) [28] are among the first to give strong validation to technical trading, or statistical based trading strategies.

BLL showed the validity of technical trading analysis by testing two of the simplest and most popular trading rules against the Dow Jones Index from 1897 to 1986. This was in direct opposition to the prevailing mindset of the time that technical trading analysis was not a strong research domain(see Malkiel (1981) [29]). Malkiel declared technical analysis was the anathema to the academic world, and its methods are patently false.

BLL results are impressive. The Variable Length Moving Average Rules yields 0.00042 daily returns for Buy signals (12% annual), -0.00025 daily returns for Sell signals (-7% annual), and 0.00067 daily returns for Buy-Sell signals (19% annual).

Skouras (2001) [30] further supports technical trading analysis by improving on the methods used by BLL. Skouras introduces an Artificial Technical Analyst to dynamically select a technical indicator rather than selecting a signal technical indicator for an entire simulation. The term Artificial Technical Analyst is defined by Skouras as an agent with the ability to change the technical indicator mid simulation, by testing multiple technical indicators on recent data and selecting the best performing. For all time frames t-N to t-1 the technical trading parameters are tested and the Artificial Trading Analyst selects the parameters that produce the highest returns.

Marshall, Young and Rose (MYR) (2006) [31] perform technical trading analysis on the 26 most widely used candlestick patterns, on the Dow Jones Index 1992 to 2002. They give a comprehensive list of the most widely used candlestick patterns and perform a comprehensive performance test of all these patterns. However, they conclude that candlestick patterns are not profitable and have no forecasting power. The candlestick patterns tested by MYR are between one and five candles. This work uses candlestick images with 28 candles. If successful, this work gives support to statistical based trading strategies, and the use of candlestick patterns.

### Computer science literature review: Financial markets predictions

Selvin, Menon, et al. (2017) propose a deep learning based architecture capable of capturing hidden dynamics to make stock market predictions [1]. Their results show evidence that the CNN is capable of identifying changes in stock market trends. Additionally they compare their CNN to a Long short-term memory network (LSTM). For their proposed methodology a CNN is identified as the best model, as it is capable of identifying inter-relation within the data.

In a related study, by Tsai, Chen, Jun-Hao and Wang 2019 [2] it is shown that traders often decide with news, fundamentals, and technical indicators, but still use their vision and experience. A CNN with candlestick images as inputs is chosen as the best tool to model a trader’s judgement. The candlestick images are preprocessed using Gramian Angular Summation Fields encoding (GASF). This format normalizes the data, reduces noise, and preserves temporal relationships. Their work cites Yang, Chen, and Yang 2019 [4] who encode time series data with three separate encoding methods, and GASF is found to be optimal. The CNN is trained and tested on EURUSD data from 2017. The output is a buy or sell signal. The model is trained on nine months of data, tested on 3 months of data, and yields an accurancy of 88% for picking the correct signal. Unlike Selvin (2017), this paper focuses on the best type of image input data, but also contributes to the overall technique that a CNN can predict financial market prices.

Kim, Taewook, Kim, and Ha Young (2019) [3] propose a fusion LSTM which combines both numeric features and image inputs. Inputs include price information, time, and multiple stock chart images to predict stock prices. Candlestick images, bar charts, and moving average line plots are all input as images into the LSTM. It is found that candlestick chart performs the best since it has more information compared with other stock chart images, and produces the highest classification accuracy of 91%. Kim et al. (2019) differs from Tsai (2019) and Selvin (2017), by using image and numeric features as CNN input.

These previous works demonstrate the ability of a CNN to make accurate predictions in financial markets. Additionally they show candlestick images work better than other image representations of the market. However they do not perform analysis as to how the CNN trades. Rather they focus on various techniques to improve model accuracy. Also, they claim to be able to predict financial markets, yet make no performance comparisons to the market itself. This work not only compares performance to the S&P 500 Index, but it utilizes feature maps to visualize what the CNN sees, by recreating images from the weights of the neural network. This is a novel contribution.

### Computer science literature review: Feature map visualizations

Feature map visualizations are used to interpret what and how a CNN has learned. The Google Brain Team research on feature visualization, notably Olah, Mordvintsev, and Schubert (2017) [9] utilizes neural network weight reconstruction to generate images. They note, in the quest to make neural networks interpretable, feature visualization stands out as one of the most promising and developed research directions.

The Google Brain Team argues that there is a growing sense that neural networks need to be interpretable to humans. If we want to understand the individual features, we can search for examples where they have high values. If we want to understand a layer as a whole, we can use the DeepDream objective, searching for images the layer finds “interesting”. Images generated by DeepDream are input, and the neurons whose activation function fired, are used to reconstruct images based on the weights. If a tree looks somewhat like a cat, the neurons which fire to classify a cat, are used to reconstruct a new image which appears like the combination of a tree and cat. While Google Deep Dream is largely used to create psychedelic images, the way it works through feature map visualization techniques, can be used for analysis of why images are classified as they are. It is seen as one of the fundamental building blocks that combined with additional tools will empower humans to understand these systems.

Along with the Google Brain Team, Zeiler and Fergus 2014 [5] are among the pioneers of feature visualization research. They argue that large CNNs have recently demonstrated impressive classification performance. However, there is no clear understanding of why they perform so well, or how they might be improved. In their paper they explore both issues. They introduce a novel visualization technique that gives insight into the function of intermediate feature layers and the operation of the classifier.

Zeiler and Fergus (2014) introduce a feature map visualization technique that reveals the input stimuli that excite individual feature maps. It also allows the observing of the evolution of features during training to diagnose potential problems with the model. Feature activations are put back into the input pixel space and re-inserted into the network. This process reveals which parts of the scene are important for classification.

In a similar study, Grun, Rupprecht, Navab and Tombari (2016) [6] acknowledge that feature visualization is a very young area of research, that begins in 2013 (see Zeiler and Fergus (2013) [5]), and Simonyan et al. (2014) [32]. They divide feature visualization into three areas: (1) input modification methods, (2) deconvolutional methods, and (3) input reconstruction methods. Input reconstruction and modification methods refer to augmenting the input images for either analysis or re-inputting into the network. Deconvolutional methods refer to generating images from the weights of the network itself for analysis.

Additionally Grun et al. (2016) introduce their own library “FeatureVis library for MatConvNet”: an extendable, easy to use open source library for visualizing CNNs. This libarary contains implementation from each of the three main classes of visualization methods. This allows feature visualizations to be created and compared from the most widely used CNN classifiers. This method gives insight into how the most popular networks classify images.

Like Zeiler and Fergus (2014) and Grun et al. (2016), Springenberg, Tobias, Dosovitskiy et al. (2014) develop their own method of feature visualization [33]. They create a deconvolutional network (decovnet) to visualize concepts learned by neurons in the layers of the neural network. They propose a new method of visualizing the representations learned by the layers of a convolutional network. This method produces more descriptive images than previously know methods. Using this method, an image is reconstructed to show the part of the input image that produces the most neuron activation output. Thus, allowing the researcher to determine which part(s) of an input image are causing neuron activation functions to fire, and shed light on why the network outputs certain values. In effect, their method produces tiny bits of images that would cause the neurons in the network to activate. Comparing these tiny bits of images to the input images gives researchers an idea on what parts of the input image would cause neurons to activate.

Similar to the Google Deep Dream approach, Dosovitskiy, and Brox (2016) [34] propose a new method to study image representations, and input these images into a CNN. The images are reconstructed from the CNN weights, to create representations. Starting with an input image of an apple, the apple image is altered to be blue. The blue apple is mis-classified as a croquet ball. The image is input into the network and the activated neurons are used to reconstruct a new image in an attempt to see what the activated neurons were looking for. The reconstructed images appear to be more like a ball, than an apple. Including a ball that appears to be in a green field. These reconstructed images give insight into why an image might have been mis-classified.

Other early feature visualization methods made strong contributions, including these by Donahue, Jia, Yangqing, et al. (2014) [35] and Yu, Yang, Bai, Yalong, et al. (2014) [36]. Donahua et al. (2014) offer a unique approach by extracting feature visualizations from a deep convolutional network, and then inputting those visualizations back into the network. Thus allowing the CNN to learn visually what segment of the image the neurons are activating on. Yu, Yang et al. (2014) admit interesting to the black-box nature on CNNs. They assert that rather than continually increasing with deeper and deeper CNN architectures, understanding the internal work mechanisms is crucial to understanding how a CNN learns. The layers of the CNN are visualized through usage of feature map visualizations. They use this technique to compare the widely used image classification CNNs: VGG and AlexNet.

Nguyen, Dosovitskiy, Clune, et al. (2016) and Nguyen et al. (2019) develop feature visualizations methods which reveal the regions in the input image cause neuron activation output, or neuron excitement [7]. They introduce a method to produce visualizations from a neural network that are synthesized from scratch. Improving our ability to understand which features a neuron has learned to detect. Not only do the images closely reflect the features learned by a neuron, but in addition they are visually interesting.

Nguyen, Yosinski, Clune, et al. (2019) [8] continued their 2016 work by implementing a feature activation technique which reconstructs images based on neuron activation for multiple layers throughout the neural network with the addition of image priors. In addition to the training images synthetic image priors are generated by augmenting training images, with the activation maximization on those images. Subsequently, when an input image is fed into the network the activation on the new image is combined with the image priors to yield an output image. This technique is able to produce a generated image that is very clear. Rather than viewing psychedelic generated images that still require some interpretation, these images generated with image priors are more clear and easier to see why an image was classified a certain way. They conclude that activation maximization techniques enable us to shine light into black-box neural networks. Improving activation maximization techniques aides our ability to understand deep neural networks.

These methods to examine a neural network through feature map visualizations are powerful. However, this work requires the ability to measure neuron excitement. Therefore, a similar approach to Nguyen, Yosinski, Clune, et al. (2019) is chosen since it gives the ability make precise measurements to changes in neuron excitement. Input images of candlesticks are combined with the neuron activation output, or neuron excitement, and used to create 2D arrays representing the neuron excitement.

## Experiment

To experiment whether an RL system can outperform the S&P 500 Index, and analyze how this is achieved, the following tests are conducted:

Test 1: An RL system utilizing a DDQN will yield higher returns than the S&P 500 Index during the testing period Jan 2, 2020 through Jun 30, 2020.

This is tested by making direct comparisons to the S&P 500 Index returns, and conducting t tests on the results to verify the consistency of the DDQN performance.

Test 2: An RL system utilizing a DDQN will demonstrate the ability to switch its attention from using all price history in 28 days of candlesticks in a candlestick image, to the most recent candles.

This is tested by generating feature map visualizations and analyzing the neuron excitement levels in the candlestick images. Regression tests are conducted to verify the change in neuron excitement in the most recent days of the candlestick image. The corona stock market crash will be used to test whether a DDQN can switch its attention based on a significant event.

An additional test is run to determine if the ability of a DDQN to yield higher returns than the S&P 500 Index, is due to its ability to switch its attention to various parts of an input image. Logistic regressions are run with the sign of daily returns as the dependent variable, and change in neuron excitement as the independent variable. This analysis will provide insight into how neuron excitement can predict positive returns.

## Methods

### Data

**Fig 5:**
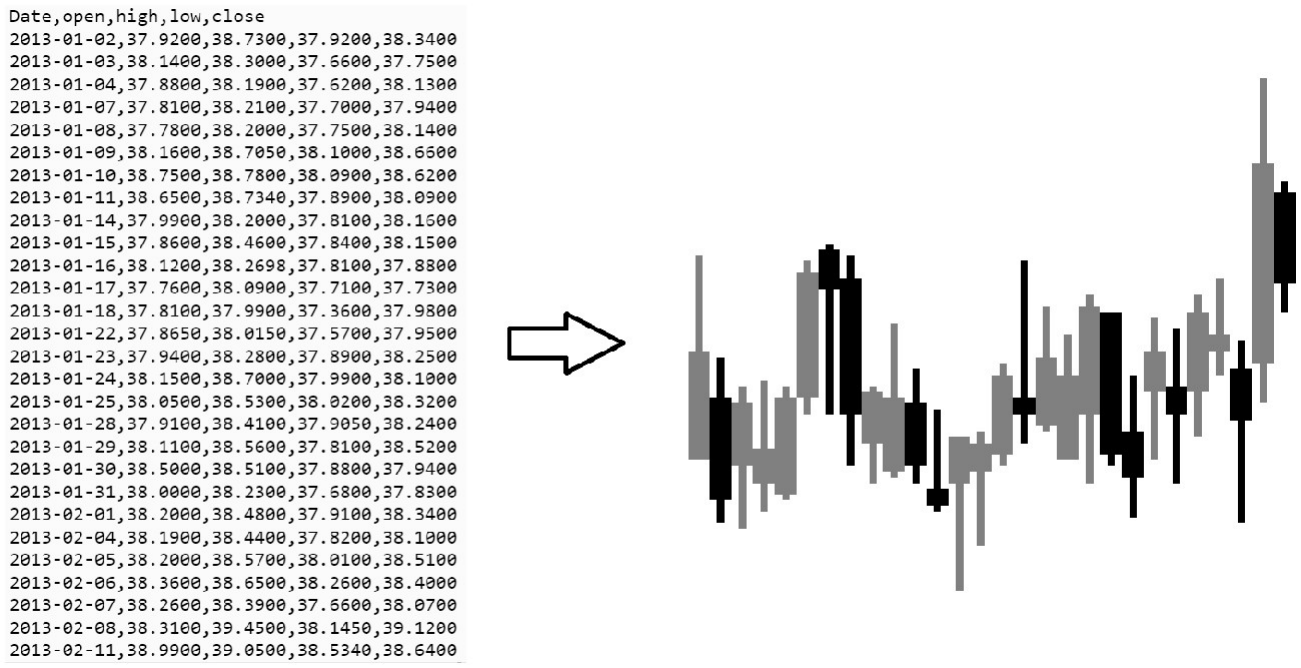
Daily high, low, open, and close stock prices are converted to a candlestick image. Gray candles indicate an upward price movement, and black candles indicate a downward price movement.

The data is pre-processed by creating custom candlestick images. To simplify the images and reduce erroneous information the body of each candle, representing open and close prices, is three pixels wide. The stick representing the high and low prices is one pixel wide. For preliminary results, each candlestick image represents 28 days of prices. A study analyzing the different number of candles per image, can be found in the results section. Gray candles indicate an upward price movement, and black candles indicate a downward price movement. The candlestick are generated for 30 stocks for the training dataset Jan, 2 2013 to Dec 31, 2019, and the testing dataset Jan 1, 2020 to Jun 30, 2020. An example candlestick image is shown in Fig 5.

### Double Deep Q-Network

**Fig 6:**
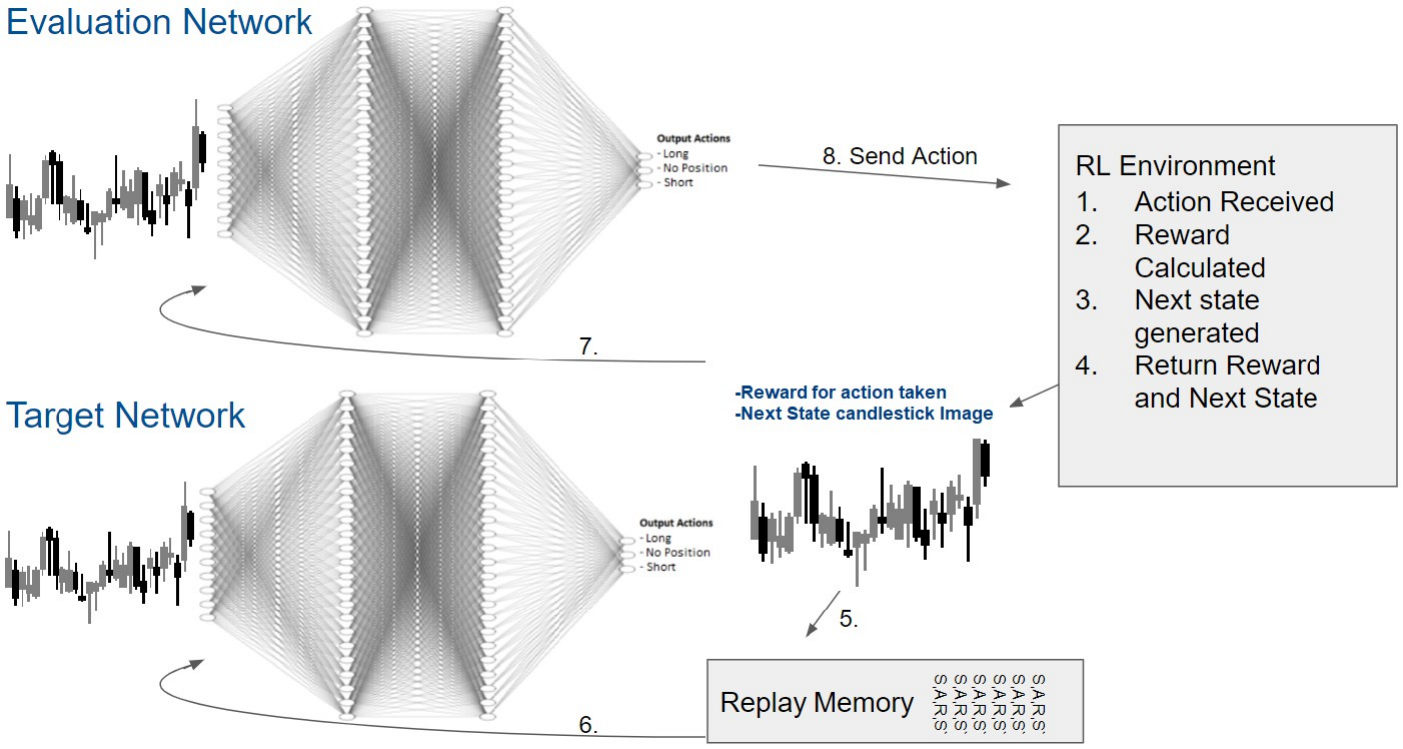
DDQN structure and workflow with RL environment. 1. An action is received by the RL environment. 2. The reward is calculated. 3. the next state candlestick image, is generated. 4. The reward and next state are returned by the RL Environment. 5. The next state is stored in the Replay memory. 6. The replay memory stores 1000 previous state, action, reward, next state observations. The target network is trained using these randomly selected previous states. Every 100 steps, the target network weights are copied to the evaluation network. 7. The next state is also directly inputted into the evaluation network. 8. The next action is outputted by the DDQN and sent to the RL Environment.

**Fig 7:**
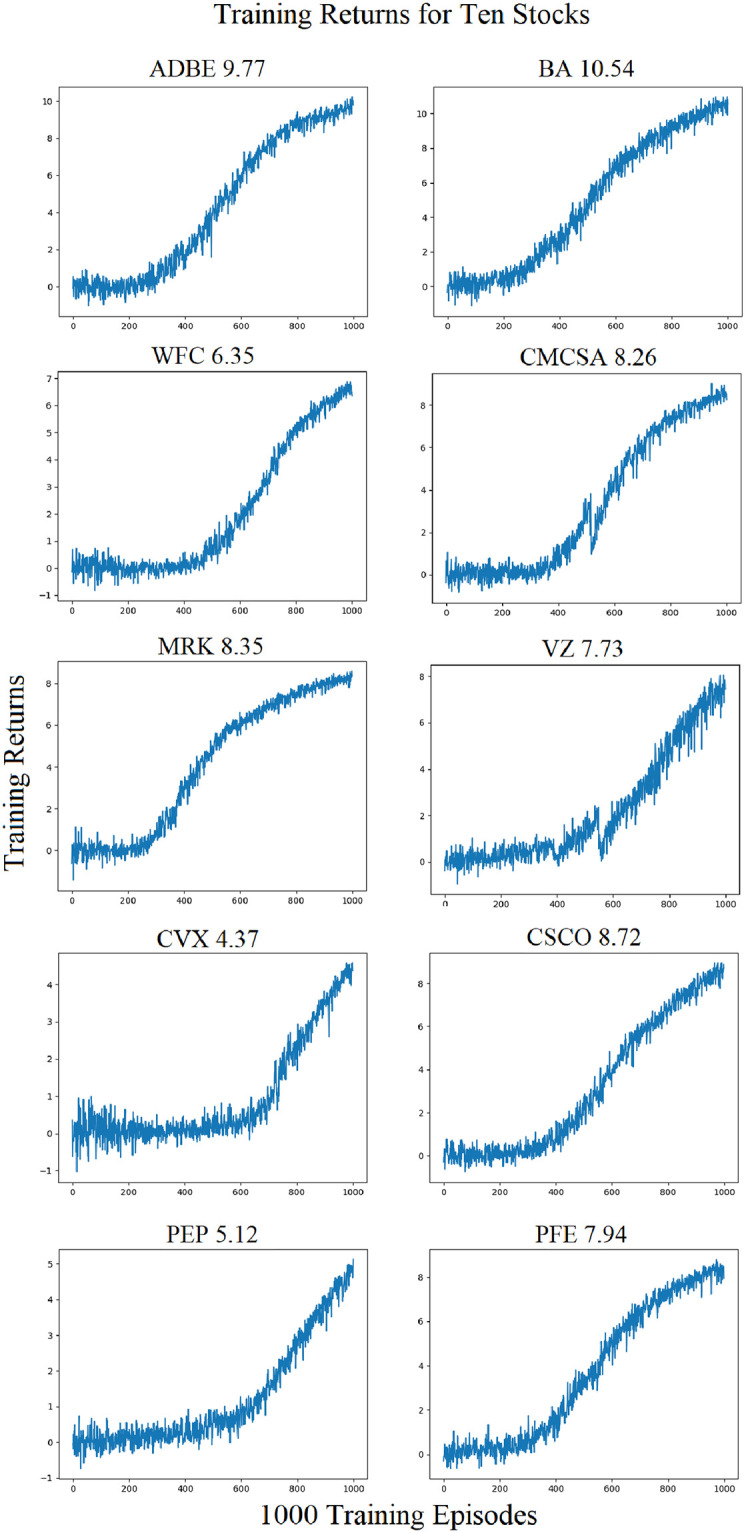
Each DDQN is able to fit a function on the training data. The training data consists of candlestick images representing stock market prices from Jan 01, 2013 through Dec 31, 2019. Final episode training rewards are shown above each training curve.

A reinforcement learning system is used with a Double Deep Q-Network for learning. The two neural networks of the DDQN are CNNs implemented in Tensorflow [37]. The architecture of the CNN consists of: input layer (84 x 84 pixels), convolutional layer (128 neurons), a second convolutional layer (256 neurons), a third convolutional layer (512 neurons), and an output layer (3 neurons for outputs long, short, or no position). The DDQN Target network is used for training on the candlestick images, and an Evaluation network for producing actions that are sent to the RL environment, implemented as an OpenAI Gym. The Evaluation network’s weights are not adjusted throughout training. The Evaluation network weights are copied from the Target network every 100 steps. The RL system training workflow is show in Fig 6. The DDQN is able to fit the training data, as shown in Fig 7.

The DDQN training rewards in the OPENAI Gym are calculated as follows:

T=a×r×N

Where:

T: Training rewards

a: action output by DDQN, action = {1, -1, 0}

r: daily returns

N: Negative Rewards Multiplier

The action space of {1, -1, 0} represents a long, short, or no position. Negative Rewards Multiplier (NRM) is used for training to increase the ability of the DDQN to take a no position action. The Negative Rewards Multiplier is a variable used to assist in training, introduced by Brim (2020) [38]. It is a constant that is multiplied, to the returns supplied from the environment. Training rewards are different from returns. Training rewards are received by the DDQN from the environment, for learning. Returns are used to calculate training rewards, and also used to compare the performance of the DDQN to the returns of the S&P 500 Index. It is used to decrease training time, as the CNN learns more quickly to output no position during training. The CNN learns to take long or short position in spite of a penalty for an incorrect output. It has no bearing on a better fit of the CNN, it simply decreases training time. Fig 7 shows the DDQN training curves for 10 of the 30 stocks. It can be seen that after approximately 400 training episodes the DDQN begins to consistently receive a positive reward form the environment. 1000 training episodes is selected as both a high enough number of training episodes to fit a CNN which is able to perform well, and also not so high as to over fit the CNN to the training data set. The training rewards on the final episode, for the 30 stocks, are shown in Table 1.

**Table 1:** DDQN training rewards on final episode, for 30 stocks.

ADBE	9.77	XOM	7.78
BA	10.54	UNH	12.39
WFC	6.35	T	7.58
CMCSA	8.26	INTC	6.64
MRK	8.35	BAC	9.78
VZ	7.73	PG	6.99
CVX	4.37	MA	9.2
CSCO	8.72	JPM	10.77
PEP	5.12	VZ	9
PFE	7.94	WMT	10.18
KO	0	JNJ	9.16
DIS	7.87	BRK.B	9.62
HD	8.21	MSFT	10.1
AAPL	10.2	AMZN	17.03
GOOGL	13.9	FB	18.5

**Fig 8:**
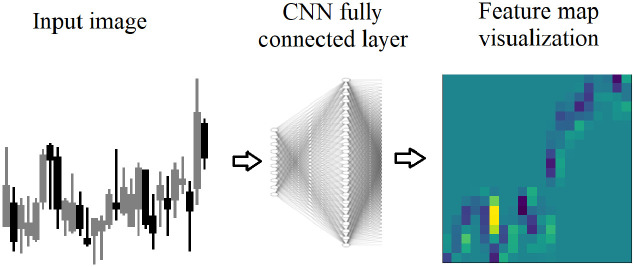
Feature map visualizations are generated from fully connected layers in the DDQN. A candlestick image is input, and each neuron in the fully connected layer receives the image. The neuron is excited on various parts of the image. The neuron is excited on various parts of the image. The excited regions are stored as a 2D array, and shown here as a heatmap. The region in yellow indicates the highest neuron excitement value on the image.

**Fig 9:**
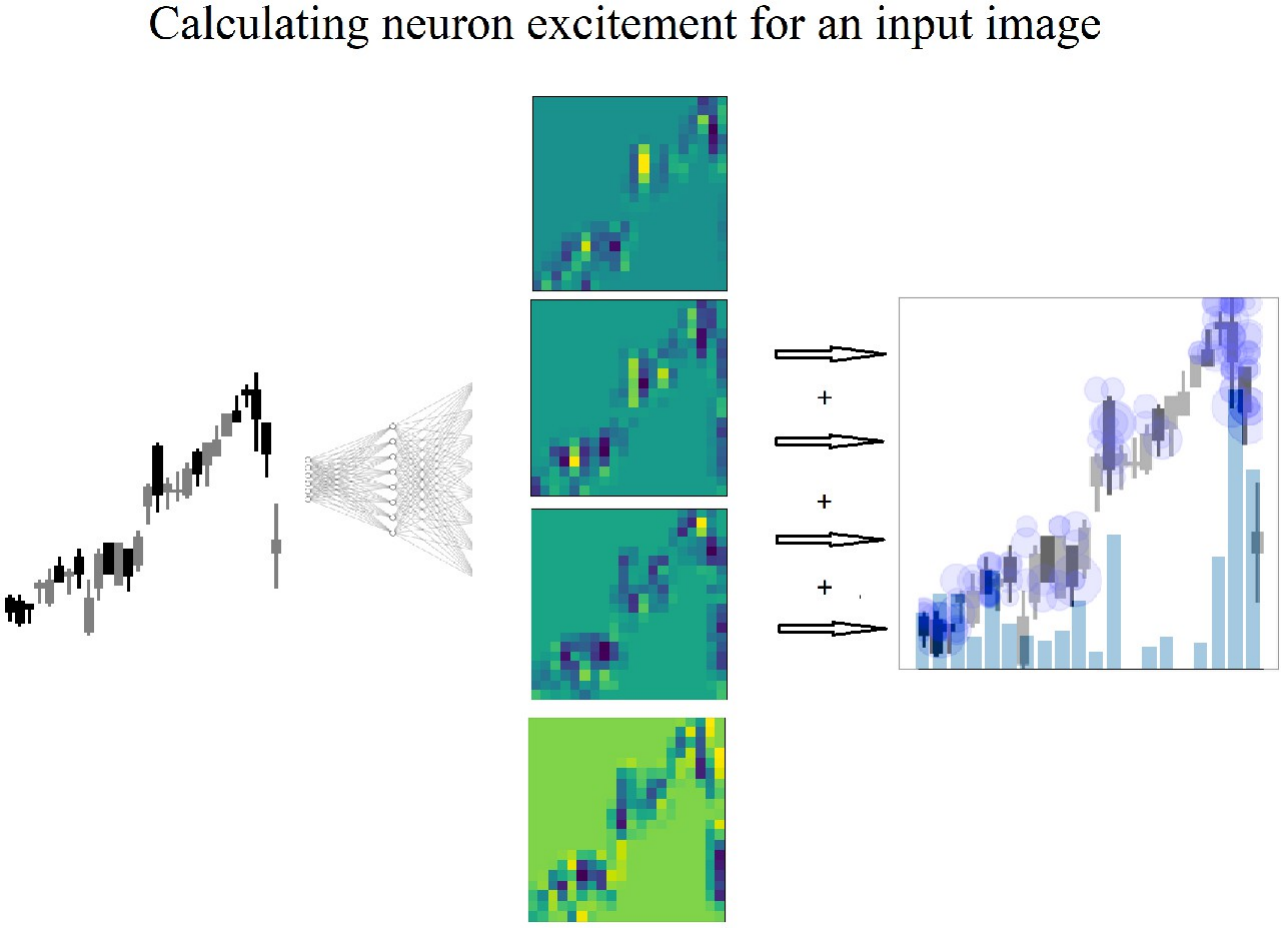
The highest level of neuron excitement, shown in yellow, for each the feature map generated from each neuron, is summed revealing the highest levels of neuron excitement by day for a single candlestick image. The size of the blue dot corresponds to the neuron excitement value. The darkness of the blue dots is caused by multiple dots overlapping. This indicates multiple neurons have produced neuron excitement on the same region. Dark blue dot clusters indicate high levels of neuron excitement. The level of neuron excitement can be seen by the overlaid blue bar chart.

After training and testing, the Evaluation network is used to generate feature map visualizations for analysis as shown in Figs 8 and 9. The OpenAI Gym Environment [24] provides a candlestick image as input into the Evaluation Network. The weights are used to create feature map visualizations which are analyzed to determine which regions of the input image are generating neuron excitement.

### Feature map visualizations

Feature map visualizations are generated from the input image and the regions of neuron excitement on the input image as show on the far right image in Fig 8. Neuron excitement is the activation output on the input image, and can be seen and analyzed in a feature map visualization. A similar approach to Nguyen, Yosinski, Clune, et al. (2019) is used in this work. Input images of candlesticks are combined with a trained CNN and used to create a 2D array of the neuron excitement.

To generate a feature map visualization, a new network is constructed with only two layers: an input layer for the input image, and a layer constructed from the weights of the fully connected layer. It would also be possible to start with a trained network and remove layers. However, utilizing Tensorflow and its built in functions, it is more suited to creating a new network and adding the layers. The input image is passed into the second layer and the neuron excitement is generated and stored in a 2D array. A feature map is generated for every neuron. In order to investigate the neuron excitement level, the highest excitement regions for each feature map are summed, as shown in the bar charts of Fig 9.

## Results and discussion

### DDQN returns vs the S&P 500 results

**Fig 10:**
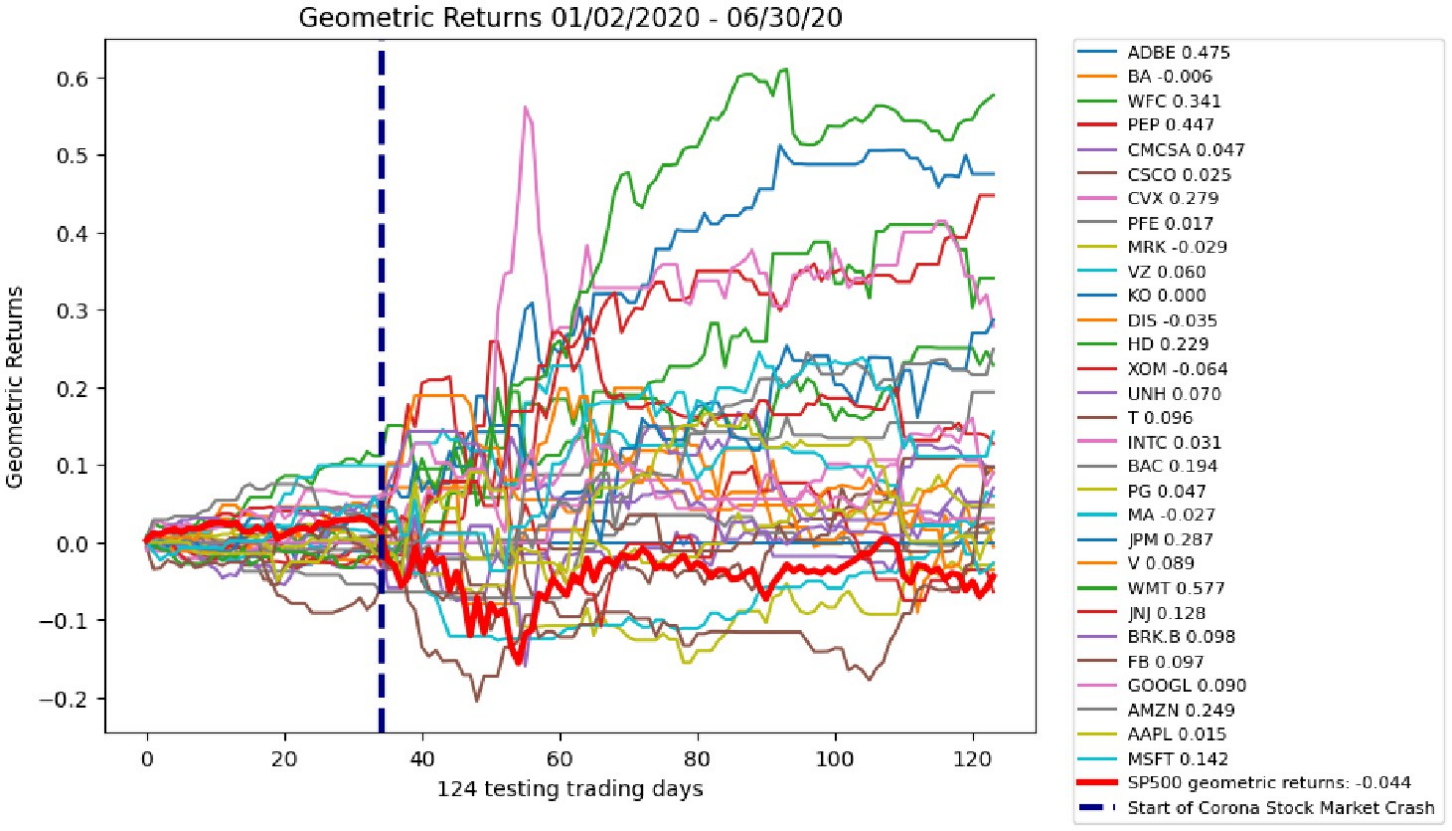
Testing geometric returns for 30 stocks. The average geometric returns is 13.2%. All but one (Exxon Mobile -6%) yield higher geometric daily returns than the S&P 500 Index. S&P 500 Index geometric daily returns are shown in the bold red line. S&P 500 Index geometric daily returns for the testing data set are -4%.

The DDQN testing results on 30 stocks yield an average of 13.2% geometric returns in 124 trading days from January 2, 2020 through June 30, 2020. The stocks with the top five geometric returns are Walmart (57.7%), Adobe (47.5%), Pepsi Co. (44.7%), Wells Fargo (34.1%) and JPMorgan (28.7%). The S&P 500 Index geometric returns during this same time yield -4% as show in Fig 10.

To verify the DDQN is able to yield higher returns than the S&P 500 Index, a T test is conducted between the geometric returns of the DDQN on testing day 124 and the geometric returns of the S&P 500 Index. The T value of -5.95 and near zero p-value of 0.0000018 clearly reject the null hypothesis that the population means are the same, indicating the difference in the DDQN returns and S&P 500 Index returns is statistically significant. This gives strong statistical evidence to support Test 1.

**Fig 11:**
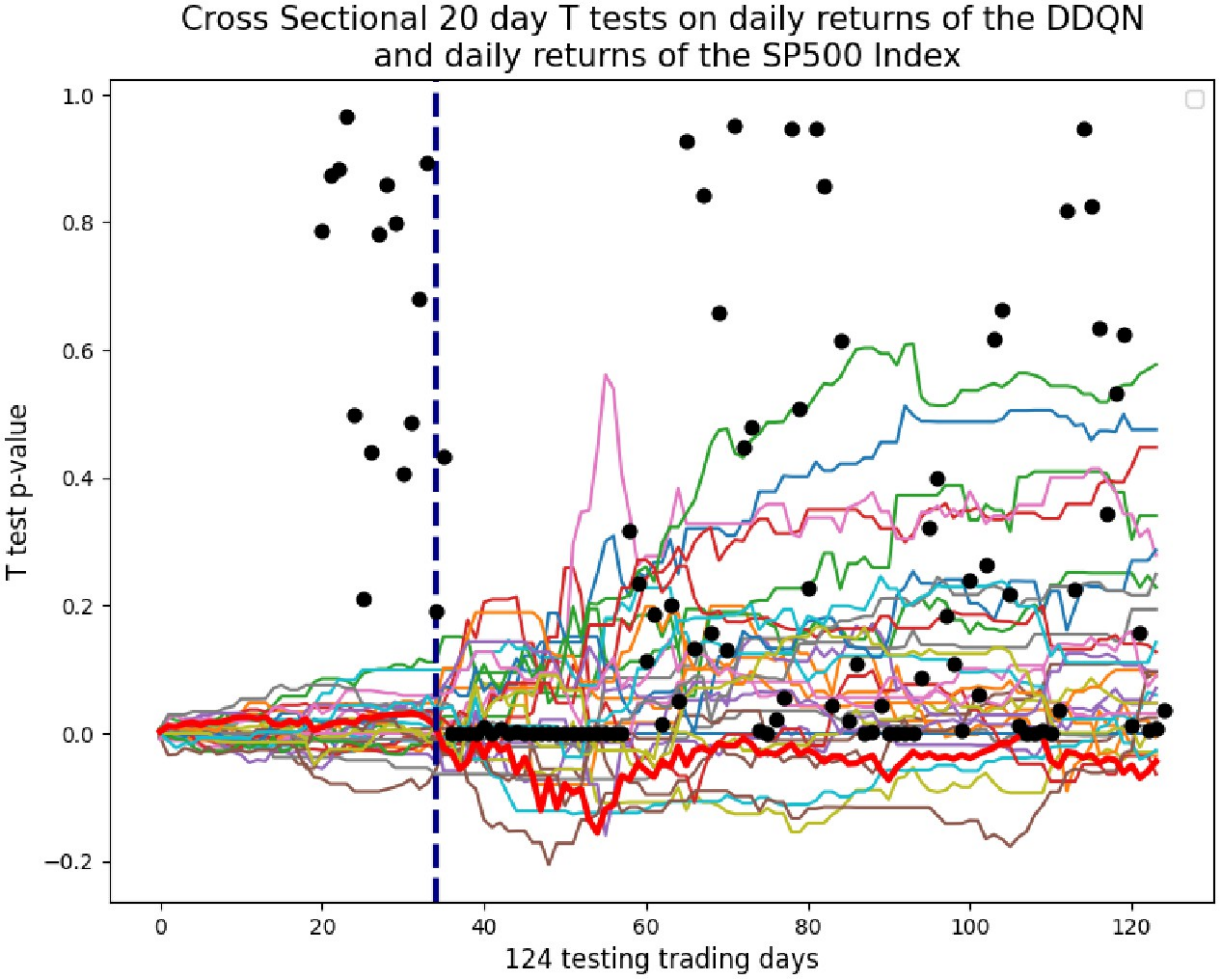
Cross Sectional 20 day T tests on daily returns of the DDQN, minus daily returns of S&P 500 Index. Each black dot is the 20 day T test p-value. The line of 22 black dots indicates that during the 22 day period following the corona stock market crash, the daily returns of the DDQN are statistically significant and different than the daily returns of the S&P 500 Index.

To further analyze the ability of the DDQN to yield higher returns than the S&P 500 Index, 20 day cross sectional t tests are conducted on daily returns of the DDQN, and the daily returns of the S&P 500 Index. The daily returns, rather than the geometric returns, allow for a day to day evaluation of the DDQN performance. Fig 11 shows the p-values of the cross sectional T tests plotted on the geometric returns. It is clear that the days immediately following the corona stock market crash are where the DDQN yields the highest returns over the S&P 500 Index. This is also the same time when the neuron excitement of the DDQN increases, as discussed in the next section.

### DDQN feature map visualizations analysis results

To determine if a DDQN can switch from using all days in a candlestick image to the most recent days, feature map visualizations are used to measure the areas of neuron excitement in candlestick images. The re-shaping of the neural network transforms the 28 candles in the candlestick image, into 20 regions. The neuron excitement values of these 20 regions is measured and analyzed. This analysis will show what part of the image the DDQN is placing attention. Additionally it can be determined if the attention is switching based on an event.

**Fig 12:**
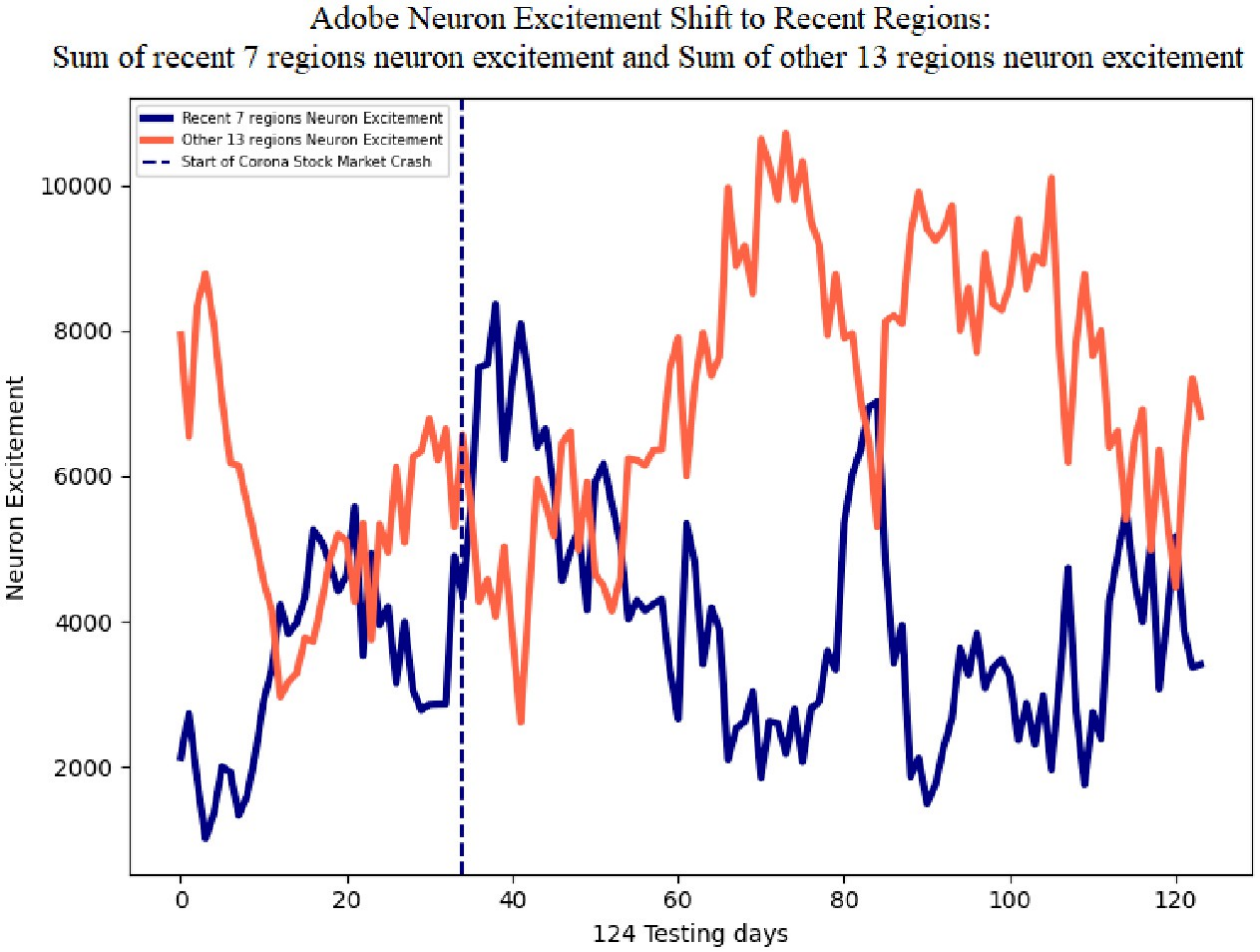
Adobe sum of neuron excitement on the recent seven regions, and the other 13 regions. Following the corona stock market crash, neuron excitement increases on the most recent seven regions and decreases on the other 13 regions.

Fig 12 illustrates Adobe sum of neuron excitement for the seven recent regions in the candlestick image in blue, and the other 13 regions in red. The average sum of neuron excitement level for the seven recent regions is 3873.07, and the other 13 regions is 6825.05. The greater average of the non-recent 13 regions is expected since these regions constitute 85% more area of the image. On day 34 of the testing data, Feb 20, 2020, the corona stock market crash begins. This event is followed by an increase in neuron excitement in the most recent seven candles and a decrease in the other 21 regions. The recent seven regions reaches a value of 8366 and the other 13 regions decreases to a value of 2610.99, indicating the DDQN is shifting its attention from all regions to the most recent regions.

**Fig 13:**
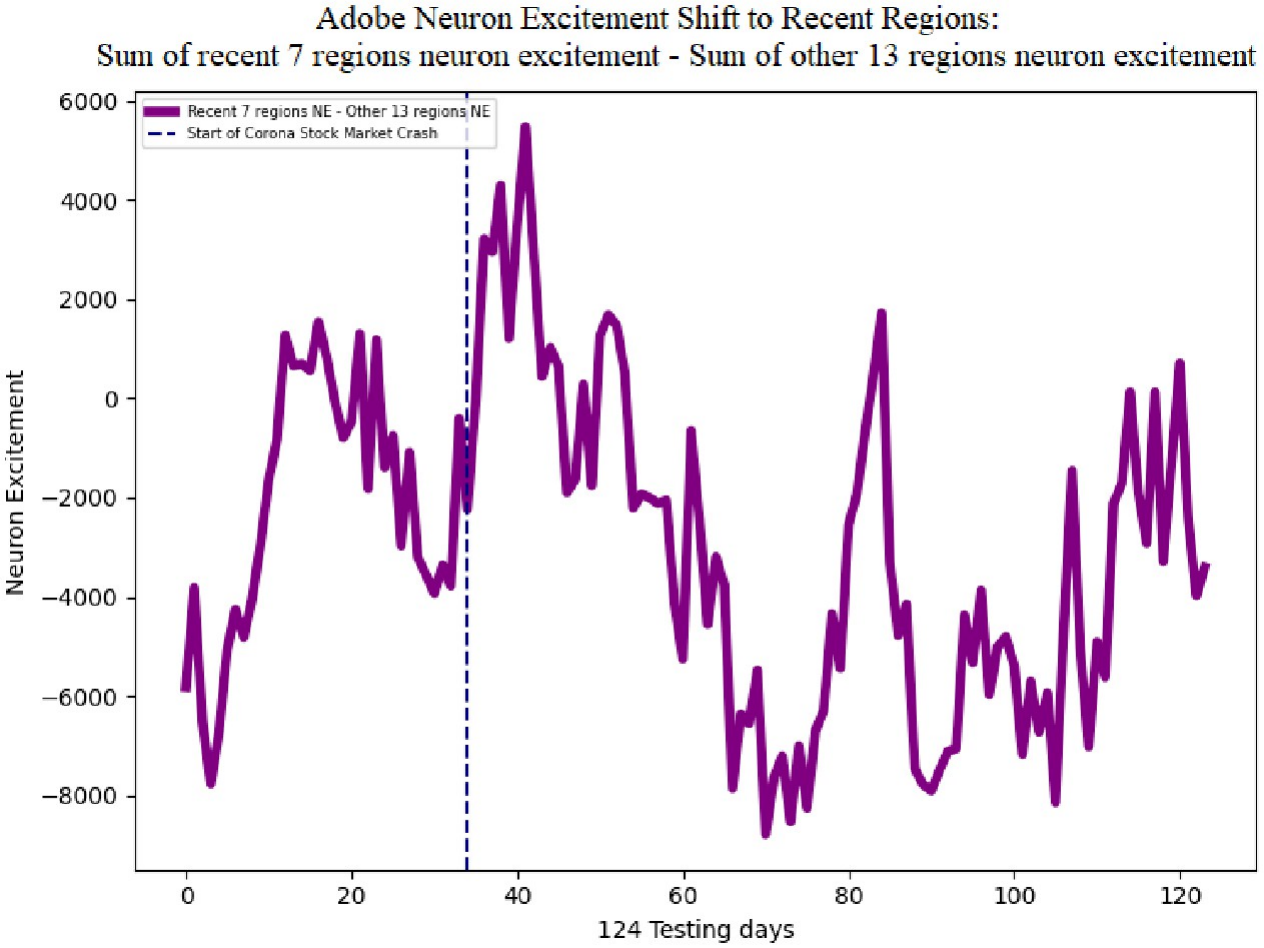
Adobe neuron excitement 7 recent regions—13 older regions. Following the corona stock market crash the sum of neuron excitement increases on the 7 recent regions and decreases on the 13 other regions.

**Fig 14:**
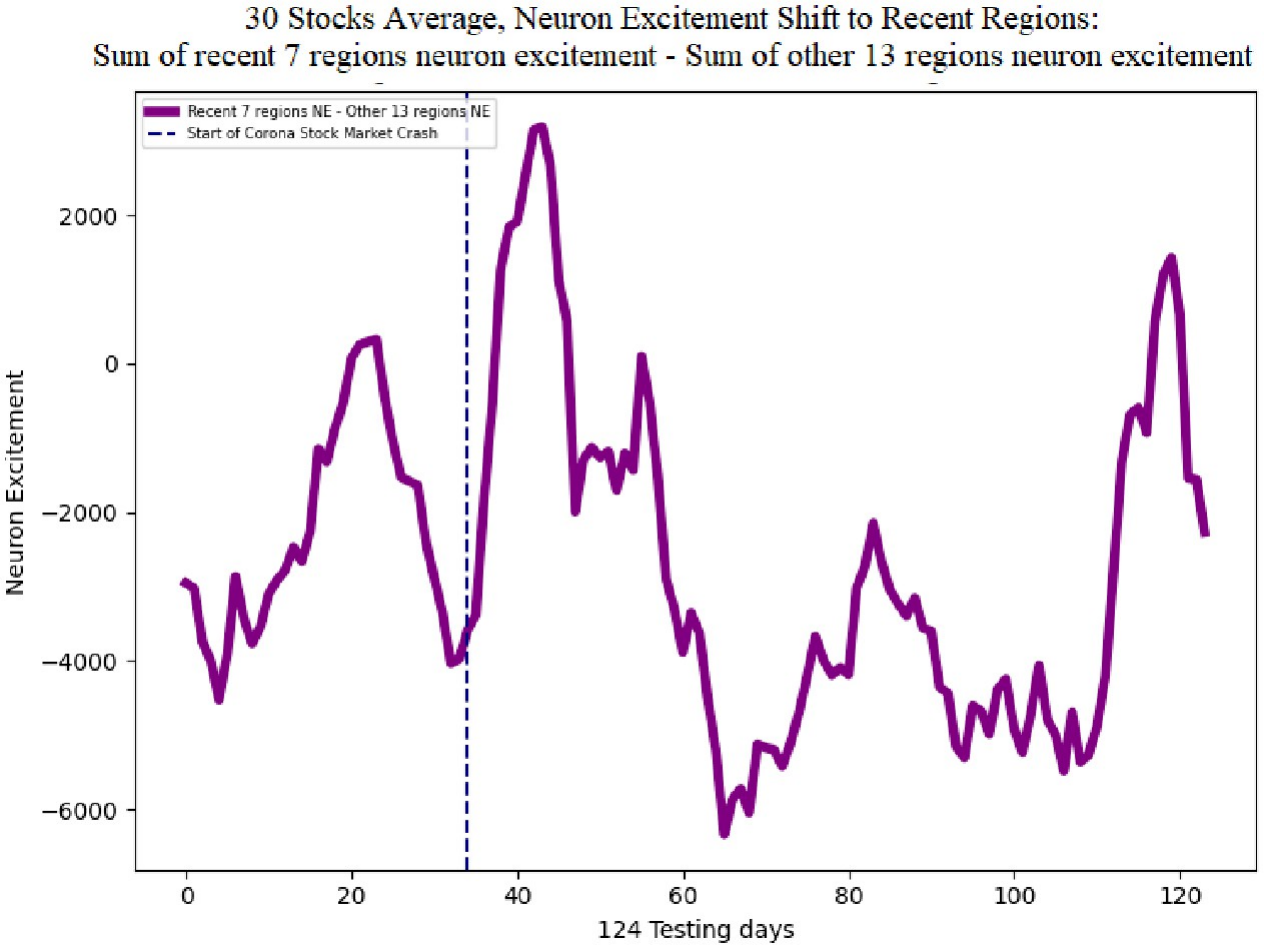
30 stocks average neuron excitement 7 recent regions—13 other regions. Following the corona stock market crash neuron excitement increases on the 7 recent regions and decreases on the 13 other regions, on average for the 30 preliminary stocks.

The single line in Fig 13 shows the difference of the sum of neuron excitement of the seven recent regions neuron excitement minus the other 13 regions for Adobe. This metric indicates a shift in neuron excitement from all regions in the image, to the most recent regions. The average difference is -2951.98. The negative value is expected since the average sum of neuron excitement of the other 13 regions is greater than the seven recent regions. Following the corona stock market crash the difference in neuron excitement increases to a maximum value of 5480.27. This behavior of the DDQN shifting attention to the recent regions in the image can also be seen in the average of all 30 stocks. Fig 14 shows the difference of the sum of neuron excitement of the seven recent regions neuron excitement minus the other 13 regions for 30 stocks. The average neuron excitement difference between the seven recent regions and the other 13 is -2643.71. Following the corona stock market crash the difference in neuron excitement increases to a maximum value of 3184.71.

This shift in neuron excitement following the corona stock market crash, for 30 stocks, shows the ability of the DDQN to switch its attention. To further analyze this behavior, and determine more exactly which regions in the image are involved, regression tests are run to statistically verify this ability of a DDQN The use of dummy variables in testing for equality between sets of coefficients in linear regressions methodology is presented in Gujarati (1970) [39]. This method allows a set of regression coefficients to be applied to each of the 20 regions in the image. The coefficients will allow for greater sensitivity testing for changes in neuron excitement. The dummy variables, of value 0 or 1, are multiplied to the coefficients in the regression. A dummy variable trap is possible here, since the regions of neuron excitement for 19 regions can be predictive of the other 1 region. To solve the dummy variable trap, one region is removed as outlined in Hirschberg (2001) [40]. Comparing the coefficients for the entire testing data set, to the 22 days following the stock market crash, will reveal the changes in neuron excitement that occur following the crash.

The first regression is run with neuron excitement as the dependent variable and the neuron excitement levels of each region as the independent variables. Dummy variables are included as coefficients to determine which regions are effecting neuron excitement. Region 1 is removed from the regression to avoid the dummy variable trap. Region 20 is the most recent region in the candlestick image, and region 2 is the oldest region. The first regression is run as follows:

$$\begin{array}{l} NeuronExcitement_{t}=\alpha +Z_{t}\beta_{2}NE.2_{t}+\\ \;Z_{t}\beta_{3}NE.3_{t}+Z_{t}\beta_{4}NE.4_{t}+Z_{t}\beta_{5}NE.5_{t}+\\ \;Z_{t}\beta_{6}NE.6_{t}+Z_{t}\beta_{7}NE.7_{t}+Z_{t}\beta_{8}NE.8_{t}+\\ \;Z_{t}\beta_{9}NE.9_{t}+Z_{t}\beta_{10}NE.10_{t}+Z_{t}\beta_{11}NE.11_{t}+\\ \;Z_{t}\beta_{12}NE.12_{t}+Z_{t}\beta_{13}NE.13_{t}+Z_{t}\beta_{14}NE.14_{t}+\\ \;Z_{t}\beta_{15}NE.15_{t}+Z_{t}\beta_{16}NE.16_{t}+Z_{t}\beta_{17}NE.17_{t}+\\ \;Z_{t}\beta_{18}NE.18_{t}+Z_{t}\beta_{19}NE.19_{t}+Z_{t}\beta_{20}NE.20_{t}+\epsilon \end{array}$$

Where:

*NeuronExcitement*_*t*_: Neuron excitement sum for all regions in a candlestick image at day t

*NE*.*Region*_*t*_: Neuron excitement value for that region in the candlestick image at day t

*Z*_*t*_: Dummy variable, 1 for each region in candlestick image, 0 otherwise.

The second regression is run to compare the coefficients of the overall testing data set, to the 22 days following the corona stock market crash. An additional dummy variable is included for the 22 days following the corona stock market crash. The 22 days following the corona stock market crash, are chosen for this test, since this is the region where the cross sectional T tests yield near 0 p-values. The coefficients in the second regression will indicate whether the recent regions have a greater effect on neuron excitement, following the corona stock market crash. The second regression is run as follows:

$$\begin{array}{l} NeuronExcitement_{t}=\alpha +Y_{t}Z_{t}\beta_{2}NE.2_{t}+\\ \;Y_{t}Z_{t}\beta_{3}NE.3_{t}+Y_{t}Z_{t}\beta_{4}NE.4_{t}+Y_{t}Z_{t}\beta_{5}NE.5_{t}+\\ \;Y_{t}Z_{t}\beta_{6}NE.6_{t}+Y_{t}Z_{t}\beta_{7}NE.7_{t}+Y_{t}Z_{t}\beta_{8}NE.8_{t}+\\ \;Y_{t}Z_{t}\beta_{9}NE.9_{t}+Y_{t}Z_{t}\beta_{10}NE.10_{t}+Y_{t}Z_{t}\beta_{11}NE.11_{t}+\\ \;Y_{t}Z_{t}\beta_{12}NE.12_{t}+Y_{t}Z_{t}\beta_{13}NE.13_{t}+Y_{t}Z_{t}\beta_{14}NE.14_{t}+\\ \;Y_{t}Z_{t}\beta_{15}NE.15_{t}+Y_{t}Z_{t}\beta_{16}NE.16_{t}+Y_{t}Z_{t}\beta_{17}NE.17_{t}+\\ \;Y_{t}Z_{t}\beta_{18}NE.18_{t}+Y_{t}Z_{t}\beta_{19}NE.19_{t}+Y_{t}Z_{t}\beta_{20}NE.20_{t}+\epsilon \end{array}$$

Where:

*NeuronExcitement*_*t*_: Neuron excitement sum for all regions in a candlestick image at day t

*NE*.*Region*_*t*_: Neuron excitement value for that region in the candlestick image at day t

*Z*_*t*_: Dummy variable, 1 for each region in candlestick image, 0 otherwise

*Y*_*t*_: Dummy variable, 1 for 22 days following corona stock market, 0 otherwise

**Fig 15:**
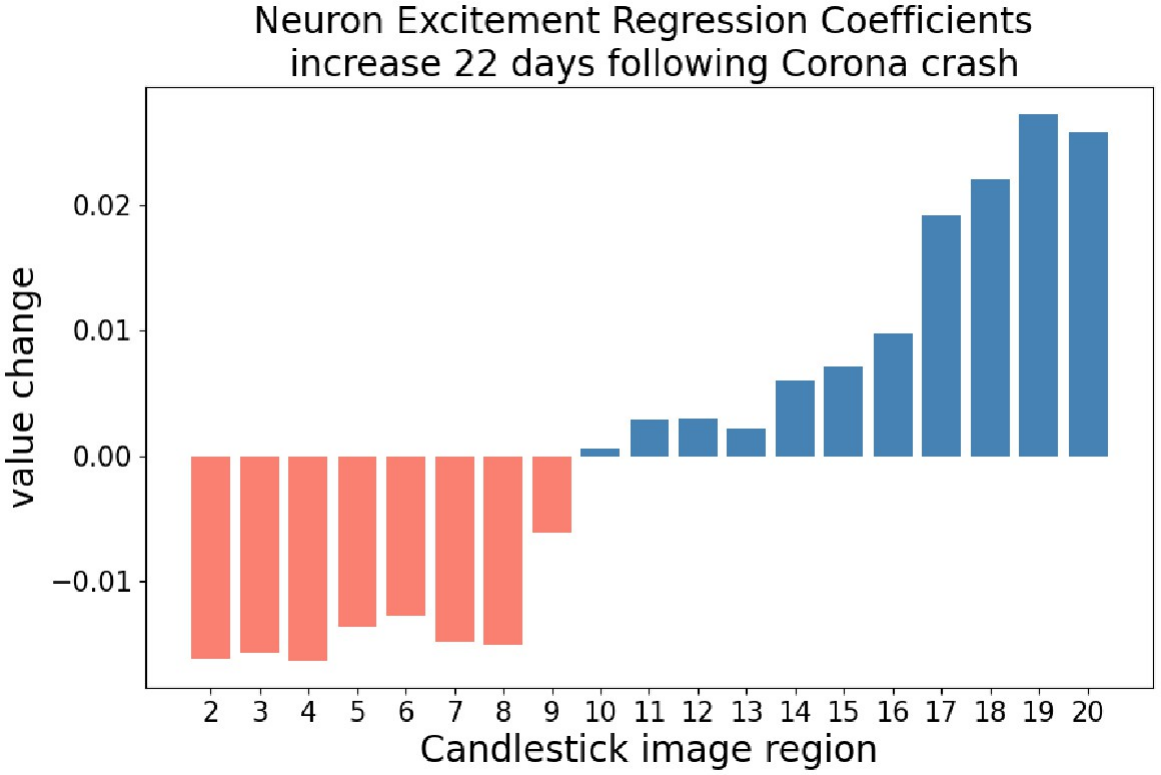
The increase in regression coefficients following the corona stock market crash. Values shown are Column B—Column A from Table 4. The neuron excitement regression coefficients for the recent regions increase while the older regions decrease. It is also notable that the neuron excitement regression coefficient with the highest increase is region 19. This region consists of the candles representing yesterday and two days ago.

Table 2 shows regression coefficients for regions 2 through 20 range from 0.0440 to 0.0518 indicating a slight increase in attention toward the recent regions for the overall test data set. A large non-zero F-statistic of 15.75 indicates volatility and significance among the coefficients. Table 3 shows the regression coefficients for the 22 days following the corona stock market crash. Regions 2 through 20 range from 0.0278 to 0.0776. A large non-zero F-statistic of 379.0 indicates volatility and significance among the coefficients following the corona stock market crash. Coefficients for neuron excitement increase for regions 9 through 20, and decrease for regions 2 through 8 as shown in Table 4. Region 19 has the greatest increase in coefficient from 0.055 to 0.0822. Fig 15 displays the values of Column 4 from Table 4.

**Table 2:** Regression results for neuron excitement. Region 20 is the most recent region, and region 2 is the oldest region. Dummy variables are used to isolate the effect of neuron excitement for each region. Region 1 is removed to avoid the dummy variable trap. The largest coefficient is region 19 (x18), the second most recent region, with a value of 0.055.

Dep. Variable:		y		R-squared:		0.004
Model:		OLS		Adj. R-squared:		0.004
Method:		Least Squares		F-statistic:		15.75
No. Observations:		70680		Prob (F-statistic):		1.56E-49
Df Residuals:		70661		Log-Likelihood:		1.11E+05
Df Model:		18		AIC:		-2.22E+05
Covariance Type:		nonrobust		BIC:		-2.22E+05
	coef	std err.	t	P>|t|	[0.025	0.975]
x1	0.044	0.001	53.288	0	0.042	0.046
x2	0.0465	0.001	56.223	0	0.045	0.048
x3	0.0479	0.001	57.962	0	0.046	0.05
x4	0.0465	0.001	56.305	0	0.045	0.048
x5	0.0472	0.001	57.048	0	0.046	0.049
x6	0.0479	0.001	57.977	0	0.046	0.05
x7	0.0479	0.001	57.919	0	0.046	0.049
x8	0.0491	0.001	59.375	0	0.047	0.051
x9	0.0495	0.001	59.882	0	0.048	0.051
x10	0.049	0.001	59.286	0	0.047	0.051
x11	0.0527	0.001	63.763	0	0.051	0.054
x12	0.0532	0.001	64.408	0	0.052	0.055
x13	0.0523	0.001	63.326	0	0.051	0.054
x14	0.0532	0.001	64.412	0	0.052	0.055
x15	0.0541	0.001	65.486	0	0.053	0.056
x16	0.0533	0.001	64.466	0	0.052	0.055
x17	0.0547	0.001	66.155	0	0.053	0.056
x18	0.055	0.001	66.592	0	0.053	0.057
x19	0.0518	0.001	62.682	0	0.05	0.053

**Table 3:** Regression results for the second regression, the 22 days following the corona stock market crash. Coefficients for neuron excitement increase for regions 9 through 20, and decrease for regions 2 through 8. The largest coefficient is region 19 (x18), the second most recent region, with a value of 0.0822. An increase of 0.0272 from the overall testing data.

Dep. Variable:		y		R-squared:		0.092
Model:		OLS		Adj. R-squared:		0.092
Method:		Least Squares		F-statistic:		379
No. Observations:		70680		Prob (F-statistic):		0.00
Df Residuals:		70661		Log-Likelihood:		8.98E+04
Df Model:		19		AIC:		-1.80E+05
Covariance Type:		nonrobust		BIC:		-1.79E+05
	coef	std err.	t	P>|t|	[0.025	0.975]
x1	0.0278	0.003	10.037	0	0.022	0.033
x2	0.0308	0.003	11.109	0	0.025	0.036
x3	0.0316	0.003	11.388	0	0.026	0.037
x4	0.0329	0.003	11.872	0	0.027	0.038
x5	0.0344	0.003	12.387	0	0.029	0.04
x6	0.0331	0.003	11.952	0	0.028	0.039
x7	0.0328	0.003	11.846	0	0.027	0.038
x8	0.0429	0.003	15.469	0	0.037	0.048
x9	0.0501	0.003	18.052	0	0.045	0.055
x10	0.0519	0.003	18.717	0	0.046	0.057
x11	0.0557	0.003	20.088	0	0.05	0.061
x12	0.0553	0.003	19.958	0	0.05	0.061
x13	0.0583	0.003	21.02	0	0.053	0.064
x14	0.0603	0.003	21.737	0	0.055	0.066
x15	0.0638	0.003	23.015	0	0.058	0.069
x16	0.724	0.003	26.103	0	0.067	0.078
x17	0.767	0.003	27.652	0	0.071	0.082
x18	0.0822	0.003	29.655	0	0.077	0.088
x19	0.0776	0.003	27.978	0	0.72	0.083

**Table 4:** Summary table of neuron excitement region regression coefficients. Column A consists of the regression coefficients for neuron excitement for all testing data. Column B consists of the regression coefficients for neuron excitement for the 22 days following the corona stock market crash. The third column shows the increase in regression coefficients following the corona stock market crash.

	A Neuron Excitement Region Coefficients All Testing Data	B Neuron Excitement Region Coefficients Corona Crash	Column B—A
region2	0.044	0.0278	-0.0162
region3	0.0465	0.0308	-0.0157
region4	0.0479	0.0316	-0.0163
region5	0.0465	0.0329	-0.0136
region6	0.0472	0.0344	-0.0128
region7	0.0479	0.0331	-0.0148
region8	0.0479	0.0328	-0.0151
region9	0.0491	0.0429	-0.0062
region10	0.0495	0.0501	0.0006
region11	0.049	0.0519	0.0029
region12	0.0527	0.0557	0.003
region13	0.0532	0.0553	0.0021
region14	0.0523	0.0583	0.006
region15	0.0532	0.0603	0.0071
region16	0.0541	0.0638	0.0097
region17	0.0533	0.0724	0.0191
region18	0.0547	0.0767	0.022
region19	0.055	0.0822	0.0272
region20	0.0518	0.0776	0.0258

It can be seen from the change in coefficients, the 22 days following the corona stock market crash, that regions 10 through 20 increase while regions 2 through 9 decrease. This indicates the neuron excitement in the 10 recent regions has a greater effect on total neuron excitement, than the older regions. The single region that most effects overall neuron excitement is region 19, representing the candles from two days ago. Both analysis of neuron excitement values, and change in regression coefficients provide evidence that a DDQN is able to switch its attention. However, these metrics do not yet confirm if the ability of DDQN to switch attention, is the reason the DDQN is able to outperform the S&P 500 Index. This is pursued in the following section.

### Logistic regressions, returns and neuron excitement results

To determine if the ability of a DDQN to outperform the S&P 500 Index is caused by its ability to switch attention from all candles in a candlestick image to the most recent, logistic regressions are run with daily returns sign as the dependent variable and change in neuron excitement as the independent variable. These regression tests will determine if positive returns are predicated by change in neuron excitement. The logistic regressions are run as follows:

$$Returns.Sign_{t}=\alpha +\beta \left(NE.Rec_{t}-NE.Oth_{t}\right)+\epsilon$$

Where:

*Returns*.*Sign*_*t*_: Daily returns sign, 1, 0 or -1

*NE*.*Rec*_*t*_: Neuron excitement recent regions

*NE*.*Other*_*t*_: Neuron excitement non-recent regions

The change in neuron excitement to the most recent regions is calculated as: recent regions—other regions. 20 logistic regressions are run for every value of recent and other regions, as the independent variable as shown in Table 5. All p-values are near zero, and all Z statistics are greater than 2 or less than -2 indicating the logistic regressions are able to successfully fit a function with daily returns sign as the dependent variable, and change in neuron excitement as the independent variable. The two regressions with the highest coefficients are the seven most recent regions at 1.5082 and six most recent regions at 1.48. This shows the change in neuron excitement in the most six or seven recent regions, is the strongest indicator of positive returns. The most negative coefficient is 13 most recent regions at -2.36. It does not indicate these regions can be used as a signal to short a stock. This simply means these regions are the least predictive to positive returns.

**Table 5:** Logistic regressions are run with the daily returns sign as the dependent variable and change in neuron excitement as the independent variable. The first and second columns show the most recent regions and other regions. Near-zero p-values and Z statistics greater than 2 or less than -2 indicate the regressions are able to successfully fit a function with daily returns sign as the dependent variable, and change in neuron excitement as the independent variable. The two regressions with the highest coefficients are the seven most recent regions at 1.5082 and six most recent regions at 1.48. Change in neuron excitement in the six or seven most recent regions of the candlestick image, is the best predictor of positive returns among those tested.

20 Logistic Regression Results					
Num of Recent Regions	Num of Other Regions	coefficient	std err	Z statistic	p-value
1	19	0.0001	0.00	26.414	0
2	18	0.2996	0.012	25.81	0
3	17	0.6402	0.026	25.017	0
4	16	0.9739	0.04	24.064	0
5	15	1.1664	0.051	22.684	0
6	14	1.5082	0.074	20.49	0
7	13	1.4838	0.087	17.073	0
8	12	1.1176	0.098	11.436	0
9	11	0.4036	0.117	3.458	0.001
10	10	-0.7143	0.129	-5.528	0
11	9	-1.6547	0.128	-12.906	0
12	8	-2.2301	0.125	-17.788	0
13	7	-2.3634	0.113	-20.967	0
14	6	-2.2663	0.098	-23.062	0
15	5	-2.0877	0.086	-24.349	0
16	4	-1.9614	0.078	-25.214	0
17	3	-1.8147	0.07	-25.773	0
18	2	-1.664	0.063	-26.34	0
19	1	-1.6709	0.063	-26.682	0
20	0	-1.6098	0.06	-26.756	0

## Conclusion

The results of Test 1 show the DDQN tested on the largest 30 stocks of the S&P 500, yield an average of 13.2% geometric returns in 124 trading days from January 2, 2020 through June 30, 2020. The S&P 500 Index returns during this same time yield -4%. On average, the DDQN yields higher geometric returns than the S&P 500 Index, by 17.2% in six months. Cross sectional t tests shows the DDQN most out performs the S&P 500 Index 22 days following the coronavirus stock market crash.

The results of Test 2 show, through the use of feature map visualizations, that neuron excitement during the 22 days following the crash, increases on the recent regions in the candlestick image and decrease on the other regions. Results also show, through the use of dummy variables in testing for equality between sets of coefficients, that a DDQN is able to switch its attention from all days in a candlestick image to the most recent days. To test whether the ability of a DDQN to outperform the S&P500 Index is due to its ability to shift its attention, 20 logistic regressions are run. The results of the logistic regressions shown that changes in neuron excitement can forecast positive returns. In this experiment, the shift in neuron excitement to the recent 7 regions is the strongest predictor of positive returns. This work not only validates statistical based trading strategies, but also provides a successful use case for candlestick images.

## References

[1] Sreelekshmy Selvin, R Vinayakumar, EA Gopalakrishnan, Vijay Krishna Menon, and KP Soman. Stock price prediction using lstm, rnn and cnnsliding window model. In *2017 international conference on advances in computing*, *communications and informatics (icacci)*, pages 1643–1647. IEEE, 2017.

[2] Yun-Cheng Tsai, Jun-Hao Chen, and Chun-Chieh Wang. Encoding candlesticks as images for patterns classification using convolutional neural networks. *arXiv preprint arXiv:1901.05237*, 2019.

[3] Kim Taewook and Kim Ha Young. Forecasting stock prices with a feature fusion lstm-cnn model using different representations of the same data. *PloS one*, 14(2), 2019. doi: 10.1371/journal.pone.0212320 30768647 PMC6377125

[4] Yang Chao-Lung, Chen Zhi-Xuan, and Yang Chen-Yi. Sensor classification using convolutional neural network by encoding multivariate time series as two-dimensional colored images. *Sensors*, 20(1):168, 2020. doi: 10.3390/s20010168 PMC6982717 31892141

[5] Matthew D Zeiler and Rob Fergus. Visualizing and understanding convolutional networks. In *European conference on computer vision*, pages 818–833. Springer, 2014.

[6] Felix Grün, Christian Rupprecht, Nassir Navab, and Federico Tombari. A taxonomy and library for visualizing learned features in convolutional neural networks. *arXiv preprint arXiv:1606.07757*, 2016.

[7] Anh Nguyen, Alexey Dosovitskiy, Jason Yosinski, Thomas Brox, and Jeff Clune. Synthesizing the preferred inputs for neurons in neural networks via deep generator networks. In *Advances in neural information processing systems*, pages 3387–3395, 2016.

[8] Nguyen Anh, Yosinski Jason, and Clune Jeff. *Understanding Neural Networks via Feature Visualization: A Survey*, pages 55–76. Springer International Publishing, Cham, 2019.

[9] Olah Chris, Mordvintsev Alexander, and Schubert Ludwig. Feature visualization. *Distill*, 2(11):e7, 2017. doi: 10.23915/distill.00007

[10] Hado Van Hasselt, Arthur Guez, and David Silver. Deep reinforcement learning with double q-learning. In *Thirtieth AAAI conference on artificial intelligence*, 2016.

[11] Takafumi Okuyama, Tad Gonsalves, and Jaychand Upadhay. Autonomous driving system based on deep q learnig. In *2018 International Conference on Intelligent Autonomous Systems (ICoIAS)*, pages 201–205. IEEE, 2018.

[12] Yi Zhang, Ping Sun, Yuhan Yin, Lin Lin, and Xuesong Wang. Humanlike autonomous vehicle speed control by deep reinforcement learning with double q-learning. In *2018 IEEE Intelligent Vehicles Symposium (IV)*, pages 1251–1256. IEEE, 2018.

[13] Han Xuefeng, He Hongwen, Wu Jingda, Peng Jiankun, and Li Yuecheng. Energy management based on reinforcement learning with double deep qlearning for a hybrid electric tracked vehicle. *Applied Energy*, 254:113708, 2019. doi: 10.1016/j.apenergy.2019.113708

[14] Bui Van-Hai, Hussain Akhtar, and Kim Hak-Man. Double deep *q*-learningbased distributed operation of battery energy storage system considering uncertainties. *IEEE Transactions on Smart Grid*, 11(1):457–469, 2019. doi: 10.1109/TSG.2019.2924025

[15] Zhang Qingchen, Lin Man, Yang Laurence T, Chen Zhikui, Khan Samee U, and Li Peng. A double deep q-learning model for energy-efficient edge scheduling. *IEEE Transactions on Services Computing*, 12(5):739–749, 2018. doi: 10.1109/TSC.2018.2867482

[16] Li Kai, Ni Wei, Tovar Eduardo, and Jamalipour Abbas. On-board deep q-network for uav-assisted online power transfer and data collection. *IEEE Transactions on Vehicular Technology*, 68(12):12215–12226, 2019. doi: 10.1109/TVT.2019.2945037

[17] Hikaru Sasaki, Tadashi Horiuchi, and Satoru Kato. A study on vision-based mobile robot learning by deep q-network. In *2017 56th Annual Conference of the Society of Instrument and Control Engineers of Japan (SICE)*, pages 799–804. IEEE, 2017.

[18] Shi Yong, Li Wei, Zhu Luyao, Guo Kun, and Cambria Erik. Stock trading rule discovery with double deep q-network. *Applied Soft Computing*, 107:107320, 2021. doi: 10.1016/j.asoc.2021.107320

[19] Li Yuming, Ni Pin, and Chang Victor. Application of deep reinforcement learning in stock trading strategies and stock forecasting. *Computing*, pages 1–18, 2019.

[20] Carta Salvatore, Ferreira Anselmo, Podda Alessandro Sebastian, Recupero Diego Reforgiato, and Sanna Antonio. Multi-dqn: An ensemble of deep q-learning agents for stock market forecasting. *Expert Systems with Applications*, 164:113820, 2021. doi: 10.1016/j.eswa.2020.113820

[21] Sutton Richard S and Barto Andrew G. *Reinforcement learning: An introduction*. MIT press, 2018.

[22] Watkins Christopher JCH and Dayan Peter. Q-learning. *Machine learning*, 8(3-4):279–292, 1992. doi: 10.1023/A:1022676722315

[23] Sutton Richard S, Barto Andrew G, et al. *Introduction to reinforcement learning*, volume 2. MIT press Cambridge, 1998.

[24] Greg Brockman, Vicki Cheung, Ludwig Pettersson, Jonas Schneider, John Schulman, Jie Tang, et al. Openai gym. *arXiv preprint arXiv:1606.01540*, 2016.

[25] Silver David, Huang Aja, Maddison Chris J, Guez Arthur, Sifre Laurent, Van Den Driessche George, et al. Mastering the game of go with deep neural networks and tree search. *nature*, 529(7587):484, 2016. doi: 10.1038/nature16961 26819042

[26] Fama Eugene F. The behavior of stock-market prices. *The journal of Business*, 38(1):34–105, 1965. doi: 10.1086/294743

[27] Fama Eugene F. Efficient capital markets a review of theory and empirical work. *The Fama Portfolio*, pages 76–121, 2021.

[28] Brock William, Lakonishok Josef, and LeBaron Blake. Simple technical trading rules and the stochastic properties of stock returns. *The Journal of finance*, 47(5):1731–1764, 1992. doi: 10.1111/j.1540-6261.1992.tb04681.x

[29] Malkiel Burton. A random walk down wall street, nueva york, 1981.

[30] Skouras Spyros. Financial returns and efficiency as seen by an artificial technicaleditor mode. analyst. *Journal of Economic Dynamics and Control*, 25(1-2):213–244, 2001. doi: 10.1016/S0165-1889(99)00074-3

[31] Marshall Ben R, Young Martin R, and Rose Lawrence C. Candlestick technical trading strategies: Can they create value for investors? *Journal of Banking & Finance*, 30(8):2303–2323, 2006. doi: 10.1016/j.jbankfin.2005.08.001

[32] Karen Simonyan and Andrew Zisserman. Very deep convolutional networks for large-scale image recognition. *arXiv preprint arXiv:1409.1556*, 2014.

[33] Jost Tobias Springenberg, Alexey Dosovitskiy, Thomas Brox, and Martin Riedmiller. Striving for simplicity: The all convolutional net. *arXiv preprint arXiv:1412.6806*, 2014.

[34] Alexey Dosovitskiy and Thomas Brox. Inverting visual representations with convolutional networks. In *Proceedings of the IEEE conference on computer vision and pattern recognition*, pages 4829–4837, 2016.

[35] Jeff Donahue, Yangqing Jia, Oriol Vinyals, Judy Hoffman, Ning Zhang, Eric Tzeng, et al. Decaf: A deep convolutional activation feature for generic visual recognition. In *International conference on machine learning*, pages 647–655, 2014.

[36] Wei Yu, Kuiyuan Yang, Yalong Bai, Hongxun Yao, and Yong Rui. Visualizing and comparing convolutional neural networks. *arXiv preprint arXiv:1412.6631*, 2014.

[37] Martín Abadi, Ashish Agarwal, Paul Barham, Eugene Brevdo, Zhifeng Chen, Craig Citro, et al. TensorFlow: Large-scale machine learning on heterogeneous systems, 2015. Software available from tensorflow.org.

[38] Andrew Brim. Deep reinforcement learning pairs trading with a double deep q-network. In *2020 10th Annual Computing and Communication Workshop and Conference (CCWC)*, pages 0222–0227. IEEE, 2020.

[39] Gujarati Damodar. Use of dummy variables in testing for equality between sets of coefficients in linear regressions: A generalization. *The American Statistician*, 24(5):18–22, 1970. doi: 10.2307/2682446

[40] Hirschberg Joe and Lye Jenny. The interpretation of multiple dummy variable coefficients: an application to industry effects in wage equations. *Applied Economics Letters*, 8(11):701–707, 2001. doi: 10.1080/13504850110042187

